# Flame emission spectroscopy of single droplet micro explosions†

**DOI:** 10.1039/d3nh00558e

**Published:** 2024-04-18

**Authors:** Jan Derk Groeneveld, Suman Pokhrel, Lutz Mädler

**Affiliations:** a Faculty of Production Engineering, University of Bremen Badgasteiner Straße 1 D-28359 Bremen Germany j.groeneveld@iwt.uni-bremen.de lmaedler@iwt.uni-bremen.de; b Leibniz Institute for Materials Engineering IWT Badgasteiner Straße 3 D-28359 Bremen Germany; c MAPEX Center for Materials and Processes, University of Bremen Postfach 330 440 Germany

## Abstract

Nanoparticles exhibit superior physical and chemical properties, making them highly desirable for various applications. Flame spray pyrolysis (FSP) is a versatile technique for synthesizing size and composition-controlled metal oxide/sulfide nanoparticles through a gas-phase reaction. To understand the fundamental mechanisms governing nanoparticle formation in FSP, simplified single-droplet experiments have proven to unravel the physicochemical mechanisms of liquid metal precursor combustions. This work introduces a novel method using flame emission spectroscopy and high-speed imaging to analyze combustion species and metal release during metalorganic single droplet combustions, with the example of the 2-ethylhexanoci acid (EHA)–tetrahydrothiophene (THT)–mesitylcopper (MiCu) precursor system. The method enables the tracing of precursor components released from droplet into the flame by spatial and temporal resolved emission tracking from combustion species (OH*, CH*, C_2_*, CS*, CS_2_*) and atomic spectral lines (Cu I). The tracking of metal emission enables the direct observation of the particle formation route, offering novel insights into the metalorganic precursor combustions. The findings of this work show a direct correlation between micro-explosions and nanoparticle formation through the gas-to-particle route. The release of copper emissions is observed with the micro-explosion event, marking the micro-explosions as the critical mechanism for the metal release and subsequent nanoparticle formation during the combustion process. The results indicate a metalorganic viscous shell formation (THT + MiCu) leading to the micro explosion. The EHA/THT ratio significantly affects the combustion behavior. Lower ratios lead to a gradual copper release before the micro explosion; higher ratios shorten the copper release and delay the micro explosion – the highest ratio results in two distinct burning stages.

## Abstract 2

Single droplet combustion analysis combining flame emission spectroscopy with high-speed imaging reveals μ-explosions as the key mechanism driving metal release and nanoparticle formation in the metalorganic-precursor combustion process.

## Graphical abstract

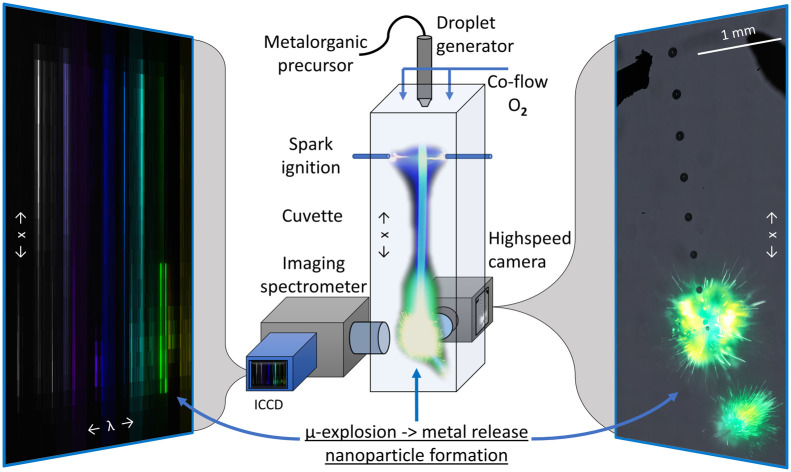

**New concepts**

We introduce a new method combining spatially and temporally resolved flame emission spectroscopy with high-speed imaging to understand the nanoparticle formation process in single-droplet combustions. Single-droplet experiments allow for analysis of the fundamental combustion mechanisms governing nanoparticle formation in flame spray pyrolysis (FSP). Previous studies on metalorganic precursor combustions relied on indirect methods for inferring the nanoparticle formation process, such as nanoparticle characterization. The here presented method allows for direct tracing of precursor-specific components, including metal release from the droplet into the flame. This provides unprecedented insights into the dynamics of precursor combustions and the nanoparticle formation pathway. The presented analysis of a copper precursor system shows the release of copper emissions to coincide with the micro-explosion events, marking these as the primary mechanism for metal release and subsequent nanoparticle formation. These results reveal a direct link between micro-explosions and nanoparticle formation through the gas-to-particle route. The uncovered microexplosion-driven nanoparticle formation process provides a new understanding of the disruptive combustion of isolated FSP droplets. These insights are crucial to designing precursor and reaction parameters to produce tailormade nanoparticles.

## Introduction

Nanoparticles possess unique physical and chemical properties due to their small size and substantial surface-to-mass ratio, making them highly attractive for various applications.^1^ Flame spray pyrolysis (FSP) produces highly functional, nanostructured metal oxide^2^ and metal sulphide nanoparticles.^3^ The FSP process allows for the specific design of high-purity and size-controlled nanoparticles.^2,4^ The method enables the utilization of almost all elements in the periodic table.^2^ Due to its high-temperature gradients and rapid quenching rate, it can synthesize metastable and multicomponent nanoparticles unable to be produced by other methods.^5^ The versatility, scalability, and single-step process make it highly demanding for a wide range of applications such as catalysis;^6,7^ energy storage,^8^ sensors,^9,10^ and biomedical applications.^11,12^

To fully understand the formation of nanoparticles in spray flames, it is essential to investigate the fundamental combustion behaviour of its smallest unit, the single droplet. The complex stochastic process of flame spray pyrolysis (FSP) is idealized as a single-droplet experiment to analyse the droplet combustion under controlled, reproducible conditions. Single droplet experiments have proven to give significant insights into the fundamental physicochemical mechanism and combustion behaviours of liquid metal precursors.^13,14^ One significant finding was the discovery of disruptive burning behaviours *i.e.* micro explosion occurring during precursor droplet combustion.^15^ The authors concluded that the micro-explosion starts with the evaporation of the high volatile low boiling point precursor. This causes the less volatile high boiling point precursor to accumulate, which inhibits mass transfer. Consequently, the droplet experiences heating due to flame shrinkage and lack of cooling through precursor evaporation. The heating causes the decomposition of the enriched metal precursor at the surface, subsequently leading to the formation of a viscous shell, causing superheating of the trapped high volatile precursor in the inner droplet and vapor nucleation, leading to a vapor bubble formation and pressure build-up inside the droplet, causing its disruption *via* a micro explosion. Precursors depicting micro explosions produce homogenous nanoparticles *via* the gas-to-particle route. In contrast, precursor combustions without micro explosion often lead to heterogeneous particles indicative of the droplet-to-particle route. These two routes were identified in FSP for the formation of inhomogeneous nanoparticles – droplet to particle route (incomplete precursor evaporation)^16,17^ and homogenous nanoparticle – gas to particle route (complete precursor evaporation)^17–19^ The single droplet combustion experiments provided an explanation for the results obtained. In FSP, the addition of carboxylic acid to the precursor was found to promote homogenous nanoparticle formation by forming volatile carboxylic metal complexes,^20^ which were also found to promote micro explosion during single droplet experiments.^14^ The involvement of micro explosions in the FSP process was initially inferred from the bimodal droplet distribution observed in metalorganic precursor combustions by Stodt *et al.*^21^ and subsequently confirmed through high-speed imaging.^22,23^ Jüngst *et al.*^24^ further proved that all metal-laden droplets in a FSP flame undergo micro explosion/disruption underlining the significance of the micro explosion for this process and the usefulness of single droplet experiments. Comparison between nanoparticles produced with FSP and single droplet experiments showed that they were almost identical, thus confirming the transferability of results gained by single droplet experiments beyond observations to the flame.^13,25^ This proven transferability between single droplet and FSP experiments allows for the economical screening and investigation of precursor–solvent combinations before scaling up to the full FSP reactor. Pokhrel *et al.*^3^ deployed single droplet combustions to identify a precursor–solvent combination that would produce metal sulphides. They successfully transferred the insight from single droplet experiments to the scaled-up production of metal sulphide nanoparticles by FSP.

In this work, investigating the single droplet combustion process is continued and expanded combining flame emission spectroscopy with established high-speed imaging. Flame emission spectroscopy is a common and relatively simple method for monitoring combustion processes.^26,27^ The technique is non-intrusive since it uses the flame as its excitation source. Flame emissions are the spontaneous emission of atoms or molecular species that were excited to an upper energy level.^28^ In single droplet combustion, emissions are caused by chemical (chemiluminescence) or thermal excitation in the reaction and flame front, respectively. The emissions during combustion can be classified into three types – atomic emission lines, molecular emission (combustion species), and broadband spectrum profiles such as black body radiation.^27^ These emissions contain valuable information about the chemical and physical processes taking place during the combustion process. Analysing these emissions, can relate properties of the reacting medium, such as equivalence ratio,^29^ temperature,^30,31^ pressure,^27^ and species concentration.^29^ In this study, the relative emission intensities were analysed and tracked throughout the combustion process to provide an *in situ* tracing of precursor components being released in the reaction front/flame front. The metal atomic spectral lines were particularly interesting, as they give a direct insight into the nanoparticle formation process, indicating when and where the metal is released during the combustion process. In the following, a setup and methodology for spatially and temporally resolved flame emission spectroscopy combined with high-speed imaging is presented and applied to a precursor system consisting of precursors with varying amounts of 2-ethylhexanoic acid (EHA), tetrahydrothiophene (THT), and mesitylcopper(i) (MiCu) to obtain a direct insight in the metal and precursor release during the combustion and micro explosion events. To the best of our knowledge, this is the first report applying this spatial and temporal resolved method to metalorganic single droplet combustions. Studies including spatially resolved flame emission spectroscopy have been reported for particle combustion,^32,33^ flame combustions^34–37^ and for pyrotechnic combustion behaviour (temporal and spatial).^38^

**Fig. 1:**
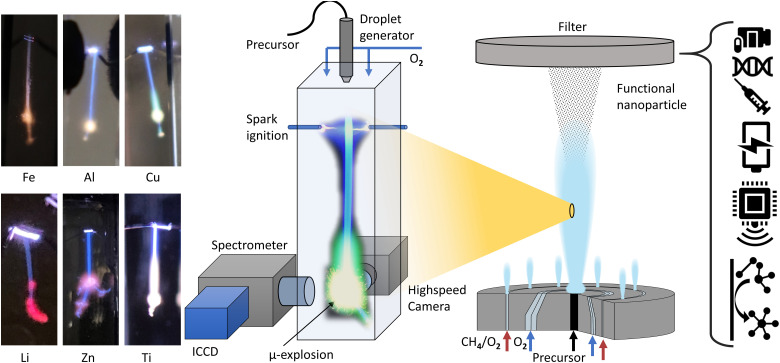
Sketch of the setup used for the experiments in this paper (center). On its right, a sketch of the FSP process. On the left, images of flame streaks from different single droplet combustions are displayed, revealing their characteristic metal-dependent flame color in connection with the micro-explosion. (Fe: ferrocene/xylene/THT, Al: Al–acetate/EHA/THT, MiCu/EHA/THT, Li: lithium nitrate/ethanol/THT, Zn: Zn–naphthenate/THT, Ti: titanium isopropoxide/THT).

Previous studies have analysed the combustion of metalorganic precursors using high-speed imaging, revealing mass transfer and phenomenological combustion behaviours/mechanism such as micro explosions.^39^*Ex situ* analysis of the produced particle and precursor chemistry was used to understand the nanoparticle formation process.^13^ These studies found the micro explosion phenomena crucial for the homogenous nanoparticle formation. However, the exact nanoparticle formation mechanism and its place during the combustion could not be directly determined, only correlated with the micro explosion phenomena. Visual observations from various metal precursor single droplet experiments and flame streak images revealing the characteristic flame colour of the metal coinciding with the micro explosion event (Fig. 1). This suggests that micro explosions are the primary mechanism for metal release and nanoparticle formation during single droplet combustion. This study aims to provide a detailed and direct analysis and observation of the link between metal release and micro-explosions, thereby deepening the understanding of this crucial mechanism for nanoparticle formation. The EHA ratio's significant influence on combustion behaviour and metal release will also be investigated using the EHA–THT–MiCu precursor system as an example.

## Methods

### Precursor preparation

Four precursors with varying EHA/THT ratios and varying Cu mol concentrations were prepared in order to analyse their influence on the multicomponent single droplet combustion. A precursor consisting of THT and EHA only was prepared for comparison. The precursors were prepared using mesitylcopper(i) (Sigma-Aldrich CAS-No: 75732-01-3) dissolved in 2-ethylhexanoic acid (Sigma-Aldrich, CAS-No: 146-57-5, 99% purity) and then mixed with tetrahydrothiophene (THT) (Sigma-Aldrich, CAS-No: 149-57-5, 99% purity) (Table 1).

Table of listed precursor combinations of different mole concentrations of Cu and ratio of THT/EHA (left) and list of complied physical properties of the used chemical compounds (right)

**Table:** 

Precursor	Cu (mol l^−1^)	EHA/THT (vol%)	EHA/THT (mol%)
P1	0.2	5/3	0.92
P2	0.1	1/1	0.55
P3	0.1	1/3	0.18
P4	0.2	1/3	0.18
P5	—	5/3	0.92

a Ref. 40.

b Ref. 41.

c Ref. 42.

d Ref. 44.

e Ref. 45.

f Ref. 46.

**Table:** 

Compound	MP (°C)	BP (°C)	VP @ RT (kPa)	Δ*H*_c_ (kJ mol^−1^)
THT	−96.13^a^	121.1^a^	2.45^a^	−2827.6 ± 0.92^e^
EHA	−59^b^	227.5^a^	0.004^d^	−4799.6 ± 1.7^f^
MiCu	184–191^c^	—	—	—

### Experimental setup

The combustion and optical analyses were performed with an in-house build single droplet combustion reactor (Fig. 1). A piezoelectric generator ejects uniform single droplets of size (∼110 μm) with a frequency of 10 Hz in a cuvette (10 × 10 × 45 mm). At the top of the cuvette, the droplets are ignited with an electric spark synchronized with the piezoelectric generator. Combustion occurs under a co-flow of oxygen (0.5 l min^−1^), regulated by a mass flow controller, at normal conditions (RT/1 atm). A photograph of the setup with descriptions can be found in the ESI.†

### Flame emission spectroscopy and highspeed imaging

The optical analysis of the droplet combustion was carried out combining high-speed imaging shadowgraphy (droplet size/combustion imaging) and flame emission spectroscopy (combustion species, atomic lines) for a spatial and temporal resolved analysis. All high-speed imaging experiments were conducted using a Phantom VEO 710 with a 3× telecentric objective and a collimated monochrome (red LED) back illumination. The limiting resolution of this setup, determined by the 1951 USAF resolution test target, is 71.8 lp mm^−1^. For each experiment, 63 single droplet combustion events were captured using highspeed imaging. The burning single droplets were evaluated for their time and size scales with a python script analysis. This script is based on an edge detection algorithm (canny) which identifies the edges of the droplets in each frame of the high-speed videos. The script further calculates an area-equivalent droplet diameter for each droplet using the number of droplet pixels.^15^ From the calculated droplet diameter, the local burning rate was calculated with the finite difference method. Flame emission spectroscopy was carried out with a UV-Nikkor 105 mm Lens (f/4.5, 0.5× WD 260 mm) imaging the flame mission of the combustion onto the slit (20 μm slit width, 6 mm height) of an imaging spectrometer (IsoPlane 160:1200 lines per mm, 300 nm blaze grating) in conjunction with a light intensified camera (ICCD) (PI-MAX4-MG: 1024F-SB-MG-18-P43, 1024 × 1024 pixel-13 × 13 μm). This setup resulted in a 50.0 nm range at a fixed central wavelength. The accuracy of the spectrometer is conservatively estimated to be ±0.15 nm, based on a 3-pixel error margin of the ICCD sensor. Flame streak images of the single droplet combustion event were taken with a digital camera for comparison with the spatially resolved emission spectroscopy.

**Fig. 2:**
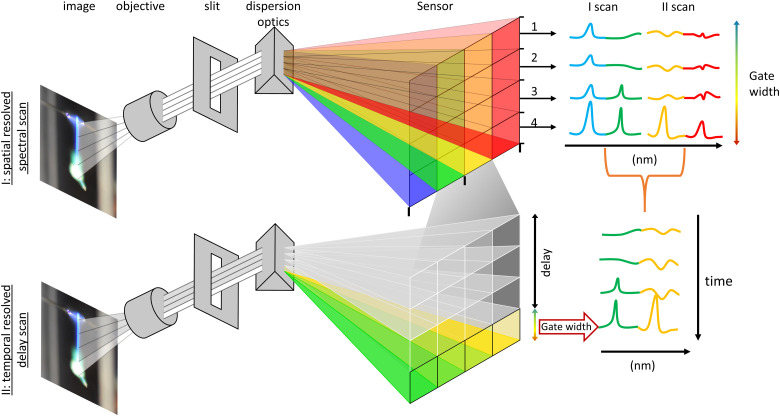
Sketch of (a) an overview of the experimental and optical setup, and (b) a depiction of the two deployed methods for the spectral analysis.

Spectral analysis was performed exploiting the repeatability and symmetry of single droplet combustion, which is (ideally) point symmetric and fully rotation symmetric along the droplet trajectory. Projecting the single droplet combustion/flame streak perfectly parallel onto the spectrometer slit makes spectral observation of the entire combustion process and averaging over several single droplet combustions possible. The alignment of the droplet trajectory parallel to the slit was accomplished mounting the droplet generator on a ball joint and an *XYZ* stage for the spectrometer/slit position. All delays for synchronizing the combustion with the optical analysis were based on the ignition spark signal, which initiates the single droplet combustion process. For a better signal-to-noise ratio and due to a remaining jitter of the single droplet combustion process, each spectral acquisition/measurement was averaged over 40 single droplet combustions integrated on the ICCD chip. Before each measurement, the ICCD gain was adjusted to the highest peak to prevent oversaturation of the sensor. The spectral analysis was conducted using two methods: first, a spatially resolved measurement was conducted for a general overview. This method resolves the entire single droplet combustion in one acquisition and allows for a rapid scan across the whole measuring range of the spectrometer (∼200 to 550 nm). The overview identifies spectral regions of interest, which exhibit the most prominent peaks of the observable combustion species and atomic spectral lines. These regions were then analysed in detail with a delay-driven temporal resolved scan to track the release of combustion species over time. The two methods are described in detail as follows and are depicted in Fig. 2:

I. The spatially resolved scan over a broad spectral range is facilitated with multitrack imaging spectroscopy. The single droplet combustion trajectory/flame streak was imaged in parallel onto the spectrometer slit. The flame emissions imaged onto the slit are dispersed perpendicular to the slit, forming a spatial-spectral image on the ICCD sensor, providing a spatial-spectral line scan along the entire trajectory of the single droplet combustion. The sensor height (*X*) corresponds to the spatial position of the light imaged onto the slit, and the sensor length (*Y*) corresponds to the spectral position of the split light (compare Fig. 2). The sensor was arranged in rows (*Y*) of 10 pixels in height (*Z*) to enhance the signal-to-noise ratio, creating independent evaluable tracks/channels – regions of interest (ROI). A scan along the spectrometer's measuring range is achieved by successively moving the scan position of the spectrometer and stitching the measurements together. Therefore, a direct a semi-temporal correlation between the flame streak image and spectral emissions regarding the flight path can be established. However, spatially spread along the droplet's trajectory, the micro-explosions event prevents a temporal analysis of its onset and metal release and allows only a spatial correlation with the flame streak image.

II. The temporally resolved emission spectroscopy is attained through a time-gated delay-driven scan. Each spectral range of interest is scanned successively with an incrementally shifted gate delay of 100 μs and constant gate width of 100 μs until the whole combustion process is covered. The recorded peaks are then compared and identified with literature values. Subsequently the spectra are background corrected, and the observable peak positions are used as starting values for Gaussian peak fitting, optimizing both position and intensity. The highest data point closest to the optimized peak positions are then extracted as the maximum peak for each spectrum. This enables tracking of maximum peak height evolution during the combustion process.

## Results

### Spatial resolved flame emission spectroscopy

**Fig. 3:**
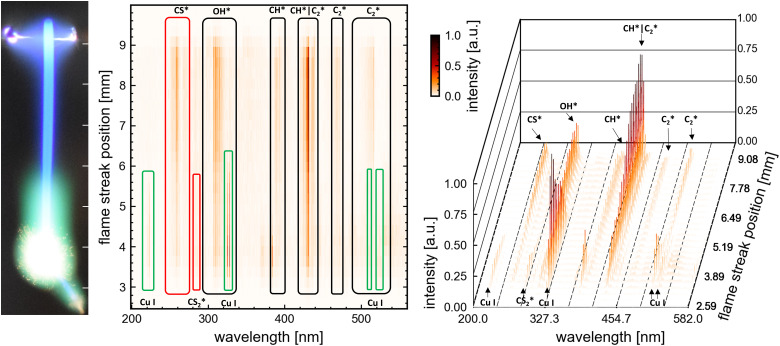
Spatially resolved flame emission spectroscopy result of P1: (left) flame streak image of an SD-combustion scaled to correlate in size with the *x*-axes of the (center) heatmap of the spectral lines observed for each ROI on the senor. Highlighted by boxes are identifiable peaks/bands of combustion species and atomic copper lines. (right) Waterfall plot for comparison and visualization of each spectral line ROI (same data as in the heatmap (center)). Representative the spectrums at 3, 6 and 9 mm can be looked up in the ESI† (Fig. S10).

The multitrack flame emission spectroscopy resulted in the observation and spatio-spectral resolved recording of Cu atomic lines and OH*, CH*, CS*, CS_2_*, and C_2_*-combustion species in the range of 200–550 nm. In detail the following emissions, indicative of the combustion of hydrocarbons and hydrocarbons containing sulphur were observed: C_2_* swan band emissions,^28,47^ OH* bands emissions,^28,48–50^ and CH* bands^28^ and peaks corresponding to CS* and CS_2_*^28,51–55^ were observed. Three spectral regions at around 225 nm, 325 nm, and 510 nm exhibit peaks of Cu I emissions.^56^ All observed signals and bands with assigned species and transitions are listed in Table 2 and are highlighted in Fig. 3. The control experiment of P5 (THT + EHA without MiCu) showed emissions for the C_2_* (swan bands), OH*, CH* and CS* bands but no peaks related to CS_2_*.

List of observable peak band positions in the combustion experiments, with compiled assigned combustion species and transition bands for OH*, CH*, CS*, CS_2_*, and C_2_* and atomic spectral lines. For each Cu line, the respective transition configuration is listed

**Table:** 

Combustion species				
Species	Transition Sys. Δ*v*-transition	Spectral region (nm)	Dom. Peak (*v*′,*v*′′) (nm)	Ref.
OH*	A^2^Σ^+^ − X^2^Π	260–350		a
	Δ*v* = +1	∼283	(1,0) 281	a
	Δ*v* = 0	∼309	(0,0) 309	a
			(0,0) 306	
	Δ*v* = −1	∼345	(0,1) 342	a
CH*	A^2^Δ − X^2^Π	∼431	(0,0) 431	b
	B^2^ Σ^+^ − X^2^Π^+^	∼389	(1,0) 389	b
	C^2^Σ^+^ − X^2^Π	∼314	(0,0) 314	c
CS*	A^1^Π − X^1^Σ^+^	240–290	(0,0) 258 (1,1) 259	d e
CS_2_^+^	B^2^Π_u_^+^ − X^2^Σ_g_^+^	282/285	282/285	d e
C_2_*	d^3^Π − a^3^Π	∼434–720		a
	Δ*v* = +2	∼435–440	—	f
	Δ*v* = +1	∼445–475	—	g h
	Δ*v* = 0	∼475–517	(0,0) 516	f g
	Δ*v* = −1	∼525–565	—	h
Mol. Term symbol 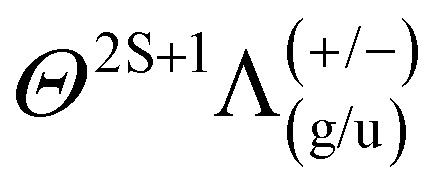	*Θ*: electronic state X ground state A,B,C… ascending states of the same multiplicity, a,b,c… different multiplicity of the ground state, S: total electronic spin angular momentum, *Λ* projection S on the internuclear axis, (g/u) inversion symmetry – even parity g and u odd-, (+/−) reflection symmetry.			

a Ref. 28.

b Ref. 57.

c Ref. 58.

d Ref. 51.

e Ref. 55.

f Ref. 59.

g Ref. 60.

h Ref. 61.

i Ref. 62.

**Table:** 

Cu-atomic spectral lines					
Specie	Peak (nm)	Configuration	Term	*J*	Ref.
Cu I	219.96	3d^9^4s^2^	^2^D	3/2	i
		3d^9^4s4p	^2^D°	3/2	
Cu I	221.46	3d^9^4s^2^	^2^D	5/2	i
		3d^9^4s4p	^2^P°	3/2	
Cu I	223.00	3d^9^4s^2^	^2^D	5/2	i
		3d^9^(^2^D)4s4p(^1^P)	^2^F	7/2	
Cu I	229.38	3d^9^4s^2^	^2^D	5/2	i
		3d^10^6p	^2^P°	3/2	
Cu I	324.75	3d^10^(^1^S)4s	^2^S	1/2	i
		3d^10^(^1^S)4p	^2^P°	3/2	
Cu I	327.39	3d^10^(^1^S)4s	^2^S	1/2	i
		3d^10^(^1^S)4p	^2^P	1/2	
Cu I	510.55	3d^9^4s^2^	^2^D	5/2	i
		3d^10^(^1^S)4p	^2^P	3/2	
Cu I	515.32	3d^10^(^1^S)4p	^2^P	1/2	i
		3d^10^(^1^S)4d	^2^D	3/2	

Visual observation of the flame streak images’ colours provides a first spectral insight into the combustion process (in the visible spectrum). The precursors P3, P2, and P4 exhibit similar flame streak patterns: a sharp blue flame streak ending in a spherical greenish-yellow micro-explosion, followed by a second micro explosion depicting the same pattern on a smaller scale. Distinct from the other precursors, P1 depicts a greenish veil around the initial blue flame streak before exploding in a spherical micro-explosion. A second smaller micro-explosion is observed, following the same behaviour. The combustion of the precursor without metal (P5 only EHA/THT) displayed a blue flame streak ending in a small orange-reddish micro explosion.

The spatially resolved multitrack-imaging spectroscopy has a direct analog spectral breakdown of the captured flame streak images, providing a broad overview of emission trends during combustion, as depicted in Fig. 3 (plots for the other precursor can be looked up in the ESI†).

The spatial resolved emission of OH*, CH*, C_2_*, and CS* are observable for all precursors with high signals at the beginning of the combustion. Their emissions spatially coaligned approximately with the blue flame streak visible in the flame streak images. For P1 and P2, these emissions show their highest emissions post-ignition, after which they decrease and peak at the end of the combustion, coinciding with the micro-explosion. For P3 and P4, these emissions show a smaller decline from moment of ignition with a small drop before peaking with the micro-explosion. Contrarily, the Cu atomic lines and CS_2_* lines are observed towards the end of the combustion for P2, P3, and P4. The CS_2_* peak and the Cu atomic spectral lines at 225 nm and 510 nm overlap with the extent of the micro explosion, as seen in Fig. 3 (see ESI† for P2, P3, P4), whereas the atomic spectral lines of Cu at 325.0 nm and 327.6 nm precede the micro explosion's extent. For P1, atomic spectral lines of CS_2_* and Cu overlap with the extent of the micro explosion and green veil visible in the flame streak image (compare Fig. 3).

Based on these findings, seven regions were selected for time-resolved flame emission spectroscopy to cover all significant observable combustion species and copper atomic lines. The seven scan regions (50 nm width) are cantered at 225 nm (Cu), 270 nm (CS* and CS_2_*), 320 nm (OH* and Cu), 390 nm (CH*), 430 nm (CH* and C_2_*), and 515 nm (Cu and C_2_*).

### Temporal resolved flame emission spectroscopy | Highspeed imaging

**Fig. 4:**
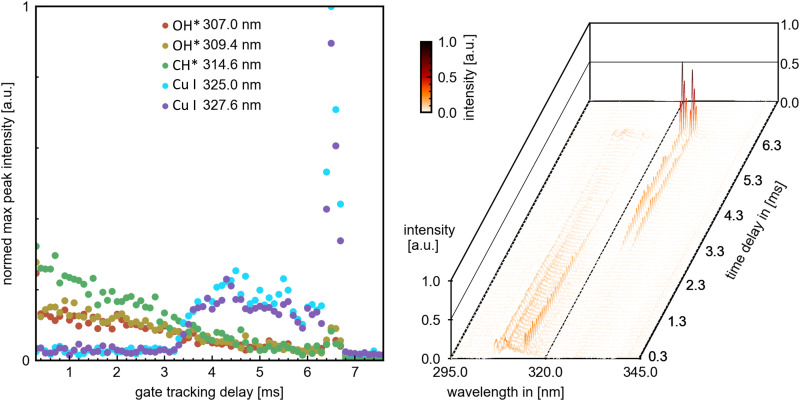
Left: Extracted peak maxima from dominant combustion species peaks and copper atomic spectral lines. Right: Overview temporal flame emission spectroscopy. Representative the spectrums at 1.0, 3.0 and 6.5 ms can be looked up in the ESI† (Fig. S11).

The combination of time-resolved flame emission spectroscopy and high-speed imaging resulted in the direct comparability of spectral emission (from combustion OH*, CH*, C_2_*, CS_2_*, and CS* species and copper atomic spectral lines) with the combustion process observed by the highspeed imaging – burning rate and combustion characteristics such as micro-explosion. This grants a direct analysis of the release mechanism of metal and other precursor components into the combustion. An example of high-speed videos of each single-droplet precursor combustion experiment (P1-P5 also named respectively) is provided in the ESI.† The four sets of combustion videos play consecutively one after another (ESI†). The temporal delay scan successfully resolved the evolution of combustion species and Cu atomic spectral lines up to the first micro-explosion. The ICCD gain was adjusted to the highest value/peak emission, causing some emission signals near a species with a higher peak intensity to scatter. An exemplary delay scan set with extracted peak maxima is depicted in Fig. 4 for the scan region around 320 nm, depicting OH* CH* and Cu emissions.

**Fig. 5:**
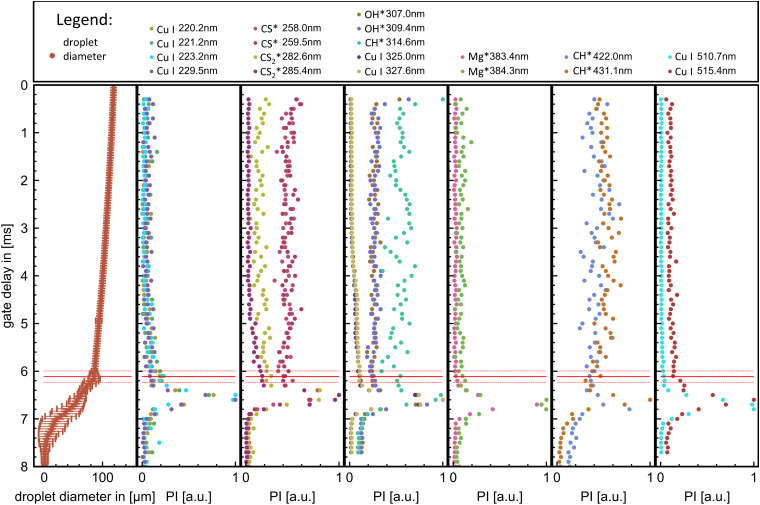
Exemplary plot of the highspeed imaging results (averaged over 63 single droplet combustions), and temporal resolved emission peaks from flame emission spectroscopy of P4. The ignition of the droplet is at a gate delay of 0 ms, coinciding with the ignition spark. Marked by the red line is the micro explosion onset with the corresponding standard deviation of the mean marked by dashed red lines.

An exemplary compilation plot of all extracted emission peak maxima with the corresponding droplet diameter evolutions derived from high-speed imaging is shown in Fig. 5 for precursor combustion P4.

The time-resolved flame emission spectroscopy and highspeed results resulted in precursor-specific combustion evolution with differentiable emission characteristics. Generally, for all precursors, the combustion process exhibits an initial steady combustion stage (burning rate) with dominant OH*, CH*, and CS* emissions—this stage transmissions for P2, P3, and P4 directly into the micro explosion event. The transition is accompanied by a decline in the burning rate and OH*, CH*, and CS* emissions and an onset and rise of CS_2_* and Cu emissions. A micro explosion followed by a second smaller micro explosion is observed for all precursors. The micro explosion event coincides with a dominant emission peak for Cu and CS_2_* emissions. Cu and CS_2_* emissions are predominantly observed for all precursors with the micro explosion. However, precursor-specific copper emission at 325.0 and 327.6 nm can be observed before the onset of the micro explosion. P1 deviates from this pattern by transitioning into a second steady burning stage dominated by CS_2_* and Cu emissions before ending in a micro explosion. A detailed differentiated description with plotted data for all precursor experiments can be looked up in the ESI† and is discussed in the following section.

**Fig. 6:**
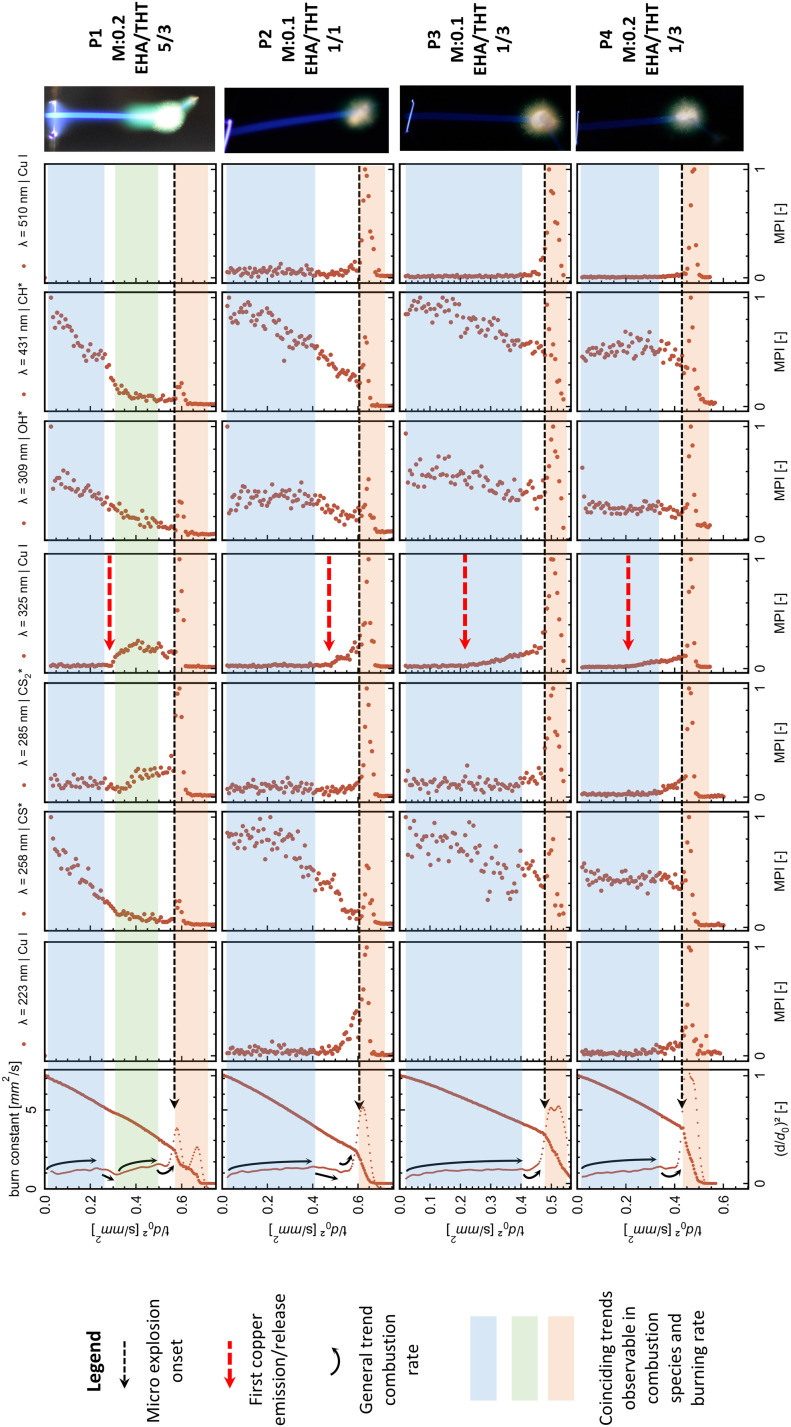
presents each tested precursor's flame emission and high-speed imaging results. Each row, with a shared time scale, begins with a graph of normalized droplet diameter and burning constant, followed by the peak emission maxima for key combustion species and copper atomic spectral lines. Burning constant trends are indicated by arrows. These trends are extended on the emission graphs by different coloured transparent fields for comparison. Micro explosion events are marked in red, with their onset shown by a dotted line and the first copper emission (Cu I 325.0 nm peaks) by a red arrow. At the right, the respective flame streak image of the precursor combustion with metal concentration and EHA/THT ratio is shown.

The evolution and onsets of OH*, CH*, CS*, and CS_2_* emissions exhibit similar trends within the same combustion species independent of the spectral region. Copper spectral lines display consistent trends within the same spectral region (around 225 nm, 325 nm, and 520 nm) while depicting different onsets and evolutions between these regions. To provide a comparative overview, the most prominent peaks for OH*, CH*, CS*, and CS_2_* emissions, along with the most significant peaks from each copper atomic spectral region for each precursor, are plotted in Fig. 6. These are presented alongside their respective droplet diameter evolutions.

## Discussion

This work introduces a novel methodology that combines flame emission spectroscopy with high-speed imaging techniques for the time-resolved analysis of combustion species and metal release during the single droplet combustion process. It enables a fundamental understanding of the combustion dynamics of metalorganic precursors. The direct tracking of combustion parameters, burning rate changes, and micro explosions, with the emissions of combustion species and atomic spectral lines, allows for time-resolved precursor component-specific insights into the combustion process.

To track copper emissions throughout the single droplet combustion process enables the time-resolved metal release from the droplet into the combustion zone. As the copper emissions are caused by the thermal excitation or reactions occurring in the flame front of the combustion zone, the copper emissions can also be used as a proxy for the time (temporally resolved spectroscopy) and place (flame streak image + spatially resolved spectroscopy) at which the nanoparticles are formed during combustion reactions.

The copper emissions for all tested MiCu–THT–EHA precursors throughout the combustion process are non-continuous. The onsets of copper emissions are observed towards the end of the combustion, collimating in strong peaks that directly coincide with the micro-explosion event (Fig. 6). This highlights the importance and interconnection of the micro explosion event for the copper release into the combustion and subsequent nanoparticle formation.

The detection of copper emissions for this precursor–solvent system shortly before the micro explosion during a steady-state combustion period (Fig. 6) provides clear evidence for the evaporation of the metal–organic precursor component. Other possible pathways, such as atomized precursor/particle facilitated by small micro explosions or puffing events, are not observed during this period. Consequently, the formation of nanoparticles *via* the gas-to-particle route in the reaction front is established and during the micro-explosion event. Another evidence for the gas-to-particle route is the green veil observable with P1 (Fig. 3 and 6). From the spectral analysis, the green colour is assigned to copper emissions, placing the nanoparticle formation in the gas phase/flame front around the droplet.

The applied and tested method provides direct observation of the particle route for precursor combustions. In contrast, before, the route was derived from the analysis of the produced nanoparticle (fine homogenous *vs.* large hollow particles).^9,13,14,17,19,39^

The above-described findings agree with the literature that correlates the formation of homogenous nanoparticles with micro explosions promoting the gas-to-particle route.^14^ This work further establishes a direct connection between micro-explosions and nanoparticle formation by gas to particle route, as the copper emission and associated nanoparticle formation are directly observed shortly before and majorly with the micro-explosion. It marks the micro explosion as the major release and formation mechanism for nanoparticles. Based on flame streak images from other precursor compositions, as depicted in Fig. 1, it is suggested that these are also applicable to other metalorganic precursor systems, which depict each metal characteristic flame colour predominant with the micro explosion event and use similar components such as EHA known to promote single droplet combustion and formation of evaporable metalorganic complexes.^13–15,63^

The tested system utilizes EHA, which forms metal carboxylate complexes, promoting micro-explosion, evaporation, and gas-to-particle route for nanoparticle formation.^14,15,17,19,39^ In this study, the combustion/emissions behaviour and metal release patterns significantly change with varying amounts of EHA. Precursors P1 and P2, with higher EHA to THT ratios of 5/3 and 1/1, respectively, exhibit similar patterns in copper emissions at 325.0/327.6 nm. The emission onset for both precursors is characterized by a sharp increase, after which P1, with the highest EHA content, enters a second steady-state combustion stage that ends in an emission peak coinciding with the micro explosion. P2, with a lower EHA ratio, transitions directly into a copper emission peak, aligning with the micro explosion. In contrast, P3 and P4, with the lowest EHA to THT ratio of 1/3, depict a continuous rise after the copper emission onset (325.0/327.6 nm), ending in a peak aligning with the micro explosion (compare from top to bottom Fig. 6). Furthermore, a higher EHA ratio seems to delay the onset of micro-explosions and the release of copper during combustion. A comparison of the initial copper emissions and micro-explosion onset between P3 and P2, both with the same metal concentration (0.1 mole Cu) but different EHA to THT ratios (1/3 and 1/1, respectively), highlights this influence of the EHA ratio delaying the initial copper release and micro explosion onsets. The observed delay in micro explosion onsets within higher EHA concentrations is in accordance with the literature.^39^

From these observations, the amount of EHA seems to influence the combustion behaviour of this system significantly. Its behaviour changed from lower EHA ratios with gradual copper release ending in a spike during the micro explosion (P3/P4) to higher ratios, causing a delayed metal release with a shortened release time before spiking with the micro explosion (P2) and with the highest ratio into a second differentiable steady-state burning stage correlating with high copper and CS_2_* emissions before spiking with the micro explosion (P1).

In more detail, Hu *et al.*^64^ investigated the influence of EHA on the nanoparticle morphology in the FSP process. They found that varying the EHA to metal (Re^3+^) molar ratio affects nanoparticle size and composition. Uniform ultrafine particles were produced with high ratios, while inhomogeneous particles were produced with low ratios. The absence of EHA results in the formation of large hollow particles. These findings are explained by the change in combustion behaviour with varying amounts of EHA observed in this work. The results of the study confirm the significance of EHA in the combustion process and the formation of nanoparticles. The observed changes in combustion patterns at the droplet level with different EHA ratios could explain these outcomes and could aid in optimizing the precursor formulation for combustion, resulting in the production of desired nanoparticles.

Rosebrock *et al.*,^15^ Li *et al.*^13^ and Li *et al.*^63^ found that the onset of micro explosion decreases with increasing metal concentration. This work confirms these findings when comparing the micro explosion onsets of P3 (0.4955 ± 0.0025 tn [μs μm^−2^]) and P4 (0.4318 ± 0.0011 tn [μs μm^−2^]) that have the same EHA to THT (1/3) ratio but different mole concentrations of copper (0.1 and 0.2 moles Cu respectively).

The observable emissions from combustion species and copper emissions can be traced back to specific precursor components. For this precursor system, copper emissions indicate the release of the metalorganic copper compound MiCu, and the CS* emissions can be directly assigned with the combustion of THT because CS* emissions are the only sulphur-related combustion species observed in the control combustion P5 consisting of THT and EHA. Conversely, CS_2_* emissions are only observed in the metalorganic precursor combustion. A correlation between CS_2_* and Cu, 325.0/327.6 emissions in metalorganic precursor combustion, is observed in the evaporation of the accumulated metal precursor and a sulphur-bearing compound at the droplet surface. This correlation is explicitly recognizable in P1 (compare Fig. 6).

THT and MiCu are known to form a metal complex [Cu 4Mes 4(THT)_2_].^65^ The structure of these complexes, determined by single crystal diffraction, is triclinic with space group *P*1. Each unit cell, with dimensions *a* = 12.907(3) Å, *b* = 20.624(6) Å, and *c* = 8.708(2) Å and *α* = 102.14(3)°, *β* = 89.64(3)°, and *γ* = 89.64(3)°, contains two formula units.^66^ RT-NMR measurements of mesityl copper dissolved in toluene revealed two sets of singlets for the aromatic and methyl protons, indicating the formation of two types of MiCu derivatives in solution: a major form (dimer) and a minor form (pentamer).^66^ The formation of tetramers of MiCu is also observed.^67^ The formation of this complex is hypothesized to occur either through the aggregation of two mesityl copper dimers^66^ or the direct interaction of a tetrameric MiCu structure^67^ in solution with THT. These complexes could be one of the accumulating species at the droplet surface. During the precursor preparation, a highly viscous gel (yellow translucent) was formed when MiCu was first mixed with THT. Conversely, a deep green stable solution was formed when THT is mixed first with EHA followed by MiCu. This gel formation might be polymerization of the metal complex, explaining the formation of a viscous shell during the combustion, triggered by the accumulation of the metal precursor and EHA (B.P: 227.5 °C) at the droplet surface *via* the preferential evaporation of the low boiling point compound THT (B.P. 122.7 °C). The preferential evaporation of THT is also observed in the CS* emission trend, which is high at the beginning of the combustion and shows a decline towards the micro explosion. Another possibility is the formation of volatile metal–carboxylic acid complexes, which are known to facilitate the gas-to-particle route and cause micro explosions in single droplet combustions^14,15,17^ or an interplay of both mechanisms regarding the significant influence of EHA on the combustion behaviour. Both hypotheses would confirm the shell formation mechanism proposed in ref. 15 for the micro explosion.

The information acquired from the emission and droplet evolution/burning rate also provides new evidence for the viscous shell hypothesis introduced by Rosebrock *et al.*^15^ as one of the primary mechanisms causing micro explosion. For P3, P2, and P4, the first segment depicts a rising burning rate ending in a plateau (highlighted by blue in Fig. 6). These segments coincide with a steady and continuous trend of OH*, CH*, and CS* emissions, which can be assigned to the combustion of THT, the low boiling point high volatile precursor component, and hydrocarbons. This part also coincides with the observed blue flame streak. The following transition, ending in the micro explosion for P3, P2, and P4, depicts a decline in the burning rate constant shortly before the micro explosion. This trend roughly coincides with a low emission reached for OH*, CH*, and CS* emissions before they peak again with the micro explosion. Conversely, a rise in Cu and CS_2_* emissions is observable. The decline before the micro explosion translates to a reduced evaporation rate caused by the vicious shell described above. The reduced evaporation and presence of a higher boiling point precursor leads to the heating of the droplet and the subsequent superheating of the remaining low boiling point THT in the inner droplet until vapor nucleation results in micro-explosions. Wang *et al.*^68^ observed a decline in the burning rate in the transition period between combustion segments and before the micro explosion onset in single droplet combustion experiments, similar to the present study. Their observation of the combustion of precursors with high boiling point different components and surfactants added directly correlates with the combustion behaviour of P1, which shows two burning segments before the micro explosion. The first segment is characterized by an increasing burning rate constant and high OH*, CH*, and CS* species emissions. The segment represents the combustion of low boiling point THT and hydrocarbons. The second segment is characterized by a rising, burning rate constant and high emissions of CS_2_* and Cu ending in a micro explosion, which is attributed to the combustion of a metal precursor and a sulphur-bearing compound (high boiling point). The two segments and micro explosion are separated by a transition period during which the burning rate decreases. This decline is attributed to the depletion of the low-boiling-point precursor and the heating of the droplet to reach the high-boiling-point component (reducing evaporation), which is in direct agreement with the observation from Wang *et al.*^68^ of single droplet combustion of a precursor with two different fuels and added surfactants (comparable to the here viscous shell formation) which also reduces evaporation and promotes the micro explosion.

## Conclusions

This work successfully introduces a new method combining flame emission spectroscopy and high-speed imaging to analyse combustion species and metal release during single droplet combustion. It provides new insights into metalorganic precursor combustion dynamics, allowing for a component-specific understanding of the combustion process at the example of the tested EHA–THT–MiCu precursor system.

I. The method allows for direct observation of particle formation routes *via* emission tracking of metal atomic spectra lines coupled with high-speed imaging during precursor combustions.

II. This work directly links micro-explosions and nanoparticle formation *via* the gas-to-particle route. The copper emission and associated nanoparticle formation are observed shortly before and primarily during the micro-explosion. Indicating micro-explosions as the primary mechanism for metal release and nanoparticle formation during single droplet combustion.

III. The EHA/THT ratio significantly impacted the whole combustion system's behaviour and copper release during combustion. EHA is shown to delay the copper release and micro explosion onset of the combustion process.

a. Lower EHA/THT ratios result in a gradual release of copper before the micro explosion (P3/P4).

b. Higher ratios delay metal release and shorten the release time before the micro explosion (P2).

c. The highest ratio leads to a second steady-state burning stage with copper and CS2 emissions, indicating the previous spiking with the micro explosion (P1).

IV. The EHA–THT–MiCu precursor system undergoes micro explosions, promoting the formation of nanoparticles *via* the gas-to-particle route. High-speed analysis and emission studies provide evidence that nanoparticle formation is facilitated by the formation of a metalorganic complex of MiCu, which leads to the formation of a viscous shell causing the micro explosion.

This study and methods provide a first detailed insight into the precursor-specific metal release and nanoparticle formation pathway. Differences in metal release patterns were observed among the tested precursors, suggesting a potential impact on the resulting nanoparticles. Further studies are warranted to evaluate this impact. The release of multiple metal components from a single precursor and the effect of different release mechanisms on the nanoparticles (homogeneous nanoparticle, solid solution, hetero-aggregates) is of particular interest. Furthermore, flame emission spectroscopy could be employed to measure temperature or equivalence ratios in the combustion process by modelling emission spectra and analysing peak ratios in the future.

## Author contributions

Jan Derk Groeneveld: conceptualization, methodology, investigation, formal analysis visualization, writing – original draft and data curation. Suman Pokhrel: funding acquisition, project administration, supervision and writing – review and editing. Lutz Mädler: conceptualization, funding acquisition, project administration, supervision and writing – review and editing.

## Conflicts of interest

There are no conflicts to declare.

## Supplementary Material

NH-009-D3NH00558E-s001

NH-009-D3NH00558E-s002
