# Supplementary material for: Flame emission spectroscopy of single droplet micro explosions
Source: Nanoscale Horiz. 2024 Apr 18;9(6):956–67. doi: 10.1039/d3nh00558e (PMC11135609; doi:10.1039/d3nh00558e)
Supplement: NH-009-D3NH00558E-s002 [file NH-009-D3NH00558E-s002.pdf]

Electronic Supplementary Material (ESI) for Nanoscale Horizons.
This journal is © The Royal Society of Chemistry 2024

# Supplementary Information

By Jan Derk Groeneveld [a,b], Suman Pokhrel [a,b,c], Lutz Mädler [a,b,c,*]

[a.] *Faculty of Production Engineering, University of Bremen, Badgasteiner Straße 1, D-28359 Bremen, Germany.*
[b.] *Leibniz Institute for Materials Engineering IWT, Badgasteiner Straße 3, D-28359 Bremen, Germany.*
[c.] *MAPEX Center for Materials and Processes, University of Bremen, Postfach 330 440.*

## Spatial resolved flame emission spectroscopy

The following plots show the spatially resolved spectral analysis of the flame streak of each precursor combustion:

P1:

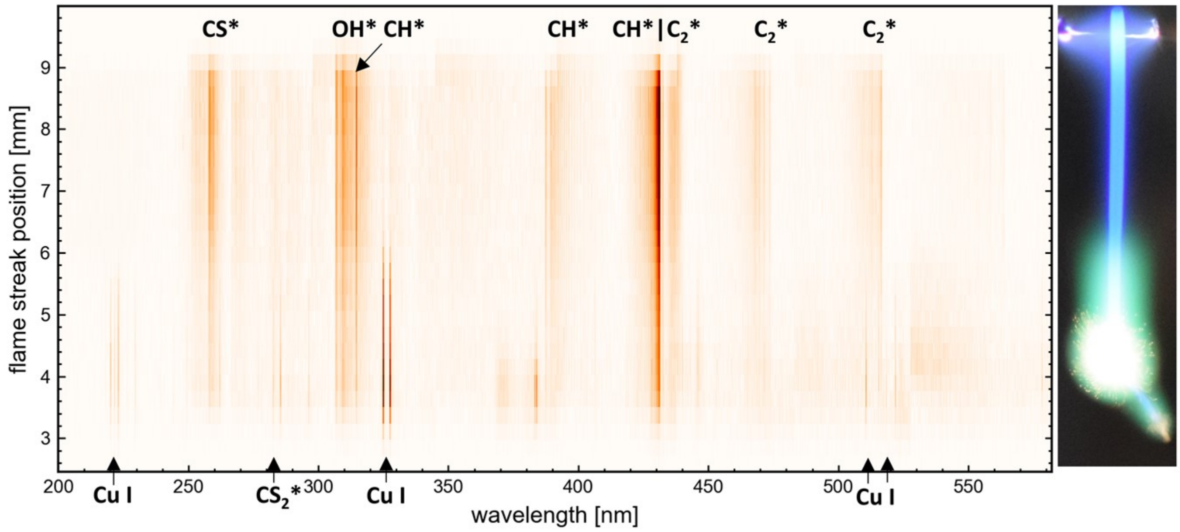


*Figure 1 spatially resolved flame emission spectroscopy results of P1: Right: Flame streak image of an SD-combustion scaled to correlate in size with the x-axes of the Left: heatmap of the spectral lines observed for each ROI on the senor. marked are identifiable peaks/bands of combustion species and atomic copper lines.*

P2:

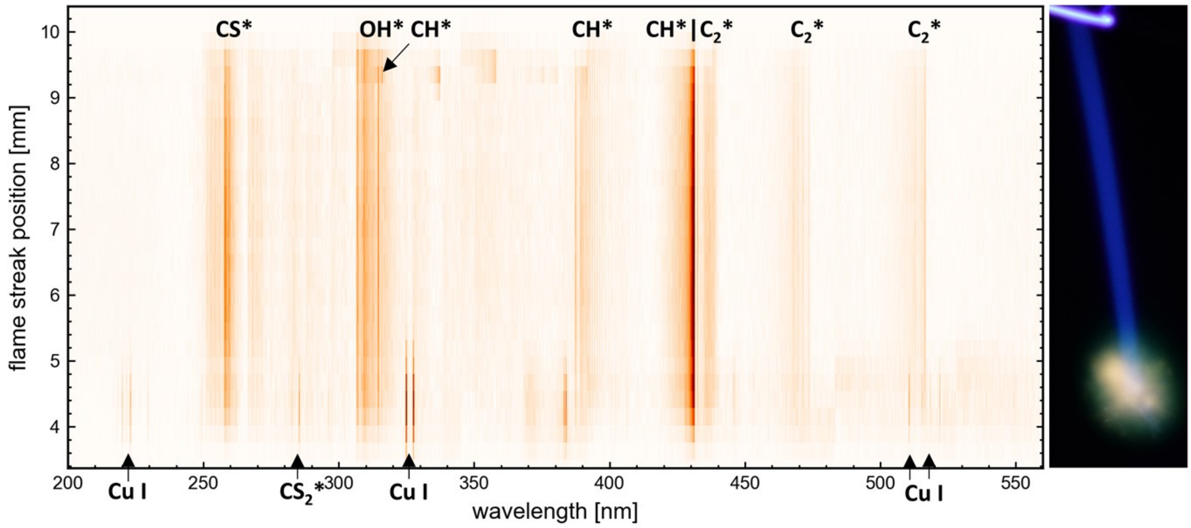


*Figure 2 Spatially resolved flame emission spectroscopy results of P2: Right: Flame streak image of an SD-combustion scaled to correlate in size with the x-axes of the Left: heatmap of the spectral lines observed for each ROI on the senor. marked are identifiable peaks/bands of combustion species and atomic copper lines.*

P3:

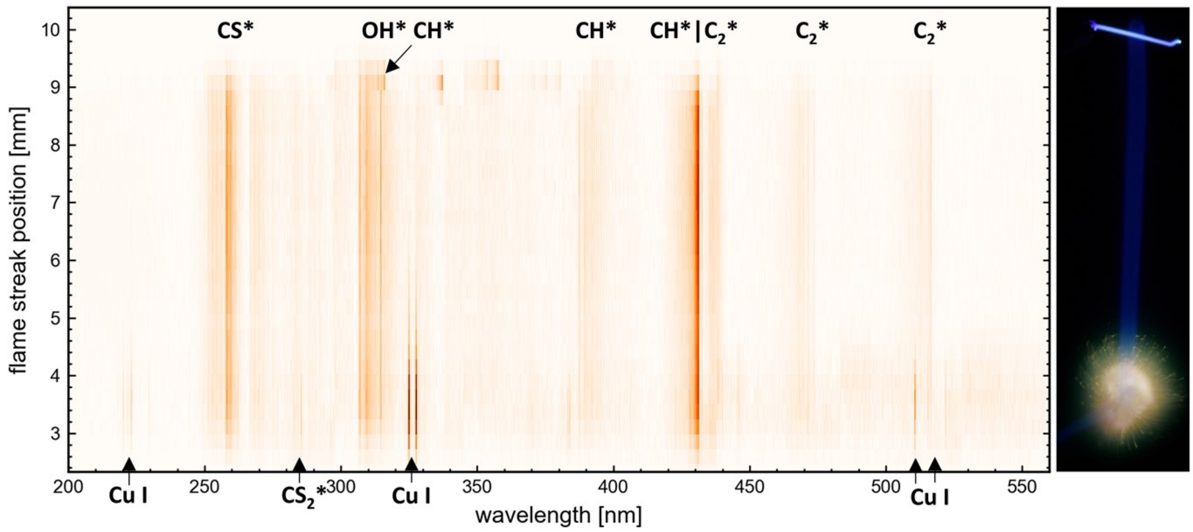


*Figure 3 Spatially resolved flame emission spectroscopy results of P3: Right: Flame streak image of an SD-combustion scaled to correlate in size with the x-axes of the Left: heatmap of the spectral lines observed for each ROI on the senor. marked are identifiable peaks/bands of combustion species and atomic copper lines.*

P4:

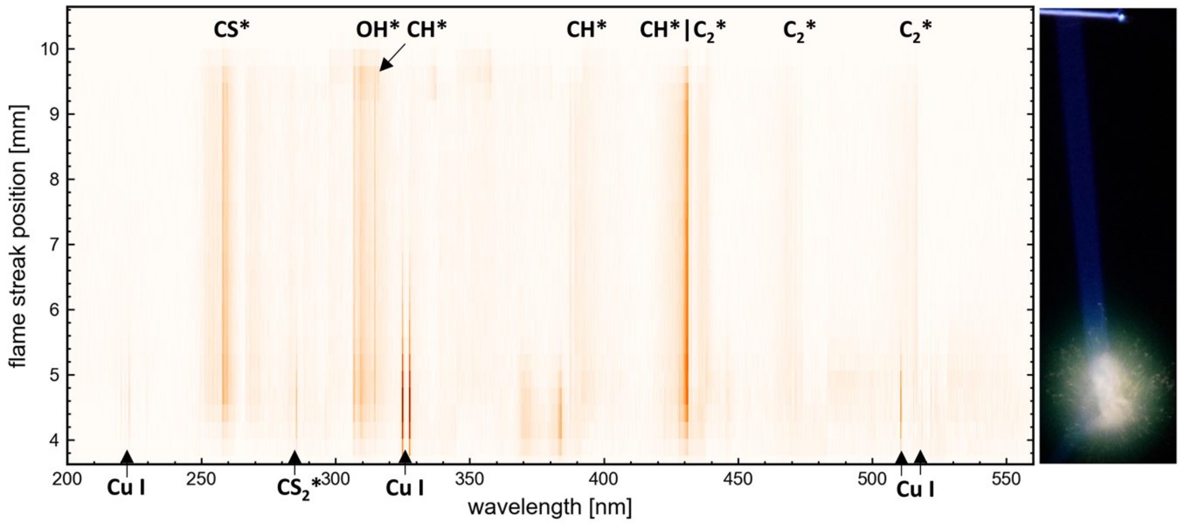


*Figure 4 Spatially resolved flame emission spectroscopy results of P4: Right: Flame streak image of an SD-combustion scaled to correlate in size with the x-axes of the Left: heatmap of the spectral lines observed for each ROI on the senor. marked are identifiable peaks/bands of combustion species and atomic copper lines.*

P5:

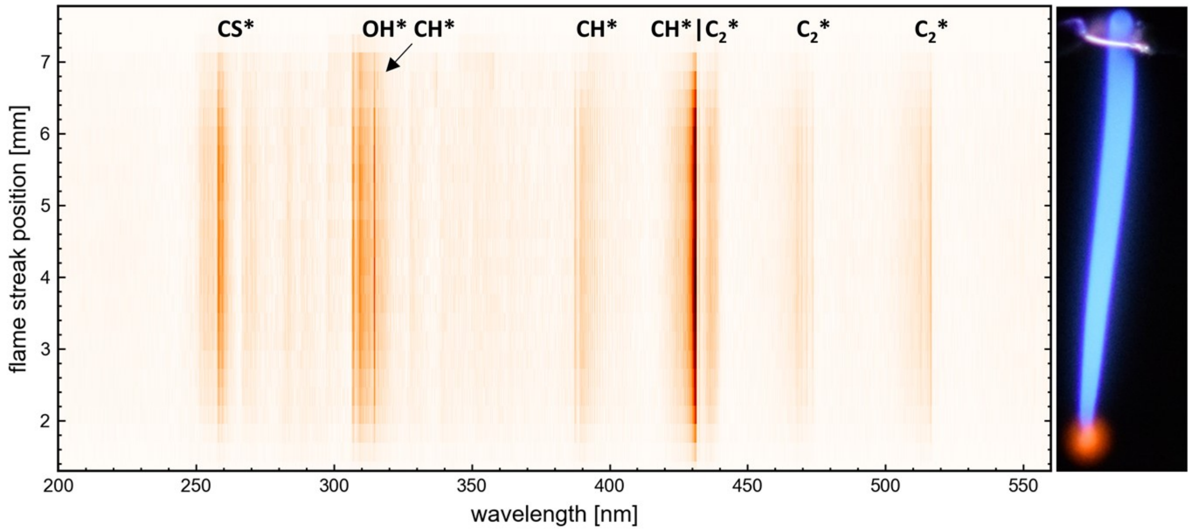


*Figure 5 Spatially resolved flame emission spectroscopy results of P3: Right: Flame streak image of an SD-combustion scaled to correlate in size with the x-axes of the Left: heatmap of the spectral lines observed for each ROI on the senor. marked are identifiable peaks/bands of combustion species.*

## Temporal resolved flame emission spectroscopy | Highspeed imaging

The following plots show the results of the temporally resolved spectroscopy and the respective highspeed imaging analysis for each precursor. Each result is described below the figure depicting the results.

P1:

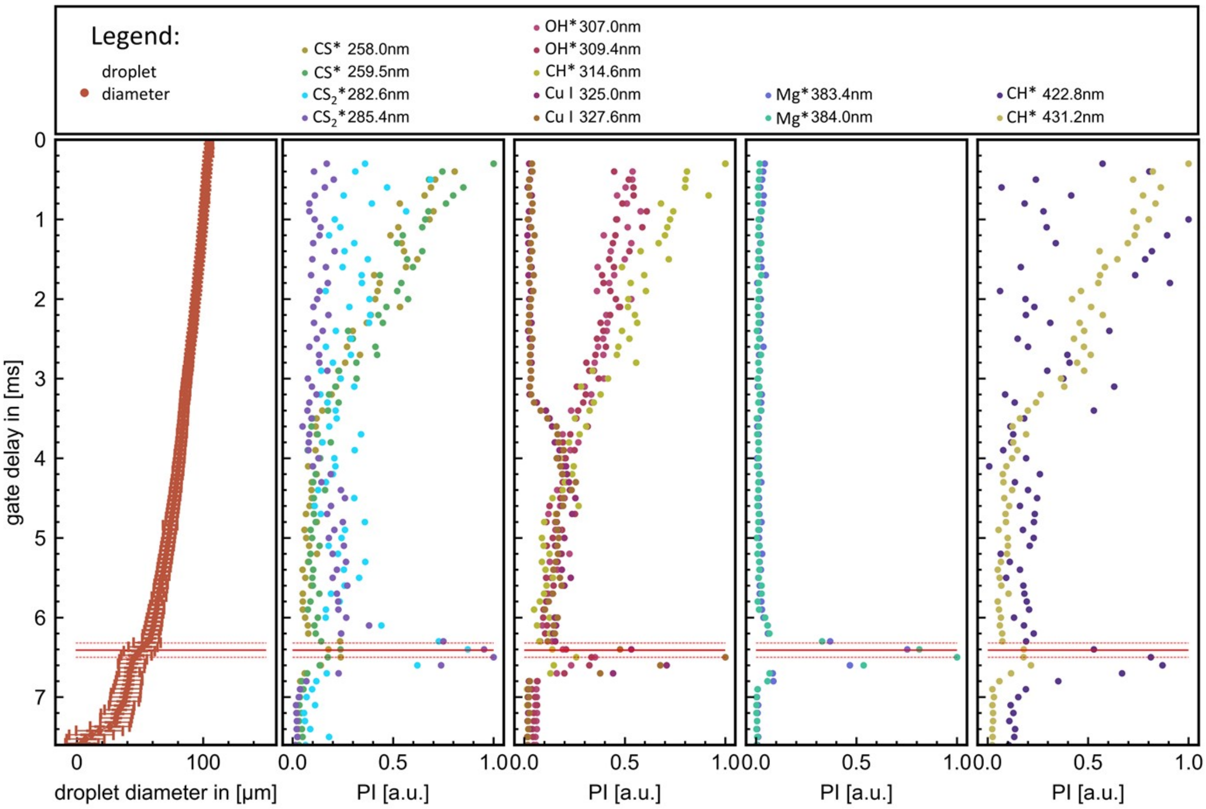


*Figure 6 plot of the highspeed imaging results and temporal resolved emission peaks from flame emission spectroscopy for P1. The ignition of the droplet is at gate delay 0 ms coinciding with the ignition spark.*

P1 shown figure 6 with 0.2 mol of Cu and a 5/3 EHA/THT ratio, four distinct segments in its droplet evolution before the first micro-explosion onset (6.410 ± 0.089 [ms] | 0.5825 ± 0.0010 tn [µs/µm2]). These segments include two main phases: the first and third segments, where burning constants increase until reaching modest maximum values (1. Segment kmax 1.27[mm2/s], 2. Segment kmax 1.53[mm2/s]). A brief transition connects these segments, marked by a decrease in the burning constant. The final segment involves a short decline and recovery

of the burning constant before the micro-explosion initiates. After the micro explosion, the remaining droplet continues to burn before exploding in a second micro explosion.

P1 (EHA/THT ratio of 5/3 and 0.2 mol Cu) displays a steep and continuous decline in CS and CH emissions from ignition to ≈ 4 ms, followed by an abrupt transition to a slow decline with low emissions to a peak with the onset of the micro explosion at 6.410 ± 0.089 ms. The intensity of OH emissions gradually decreases from ignition, and reaches a minimum just before the onset of micro explosion at 6.410 ± 0.089 ms, followed by a spike in emissions with the micro-explosion. The copper signals at 325.0 and 327.6nm show a steep increase beginning at ≈ 3.7 ms, reaching a maximum before gradually decreasing and culminating in a strong emission peak from the onset of the micro-explosion. The delay scan at 225 nm and 510 nm failed due to droplet instability occurring before the experiment was completed. CS2 emissions exhibit a similar trend to Cu 325.0 and 327.6 emissions with a slight delay. They exhibit a steep rise from ≈ 4 ms, then gradually increase, until depicting a strong peak from the onset of the micro-explosion.

P2:

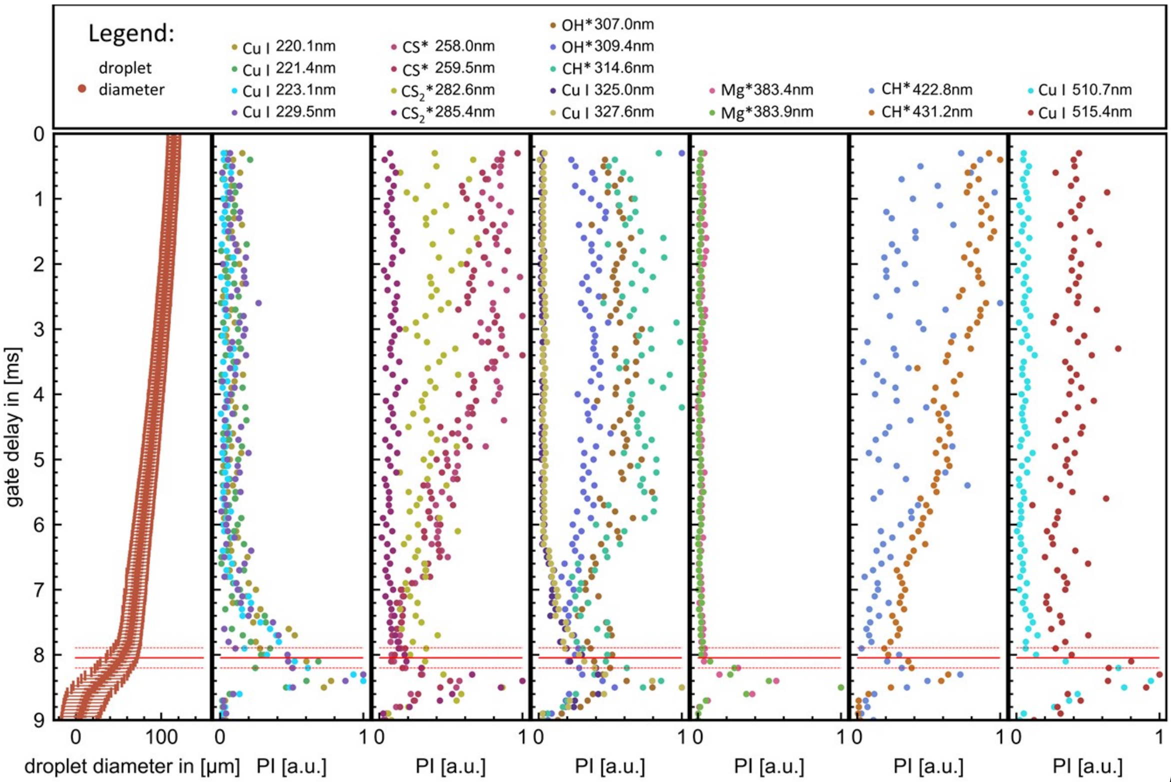


*Figure 7 plot of the highspeed imaging results and temporal resolved emission peaks from flame emission spectroscopy for P2. The ignition of the droplet is at gate delay 0 ms coinciding with the ignition spark.*

The combustion pattern of P2 shown in Figure 7 exhibits two distinct segments before the onset of the first micro explosion at 8.045 ± 0.155 [ms]. The first segment is characterized by an increasing burn rate, reaching a maximum of 1.39 [mm2/s]. The second segment starting shortly before the micro explosion onset is characterized by a brief decrease in burn rate, followed by a subsequent increase. A second micro explosion is observed shortly after the first micro explosion.

P2 initially shows nearly constant emission trend for CS and OH. At around ≈ 4 ms, the emission intensity shifts to a decaying pattern until reaching a low point just before a sharp emission peak with the micro explosion is observed. The CH peaks show a decline from ignition, and reach a low point shortly before a prominent emission peak coinciding with the micro-explosion. The copper signals at 325.0 and 327.6 nm rise asymmetrically from ≈ 6.2 ms, culminating in a strong peak coinciding with the onset of the micro-explosion. Both copper peaks at 510.7 nm and CS2 peaks rise shortly before terminating in a strong peak coinciding with micro explosion.

P3:

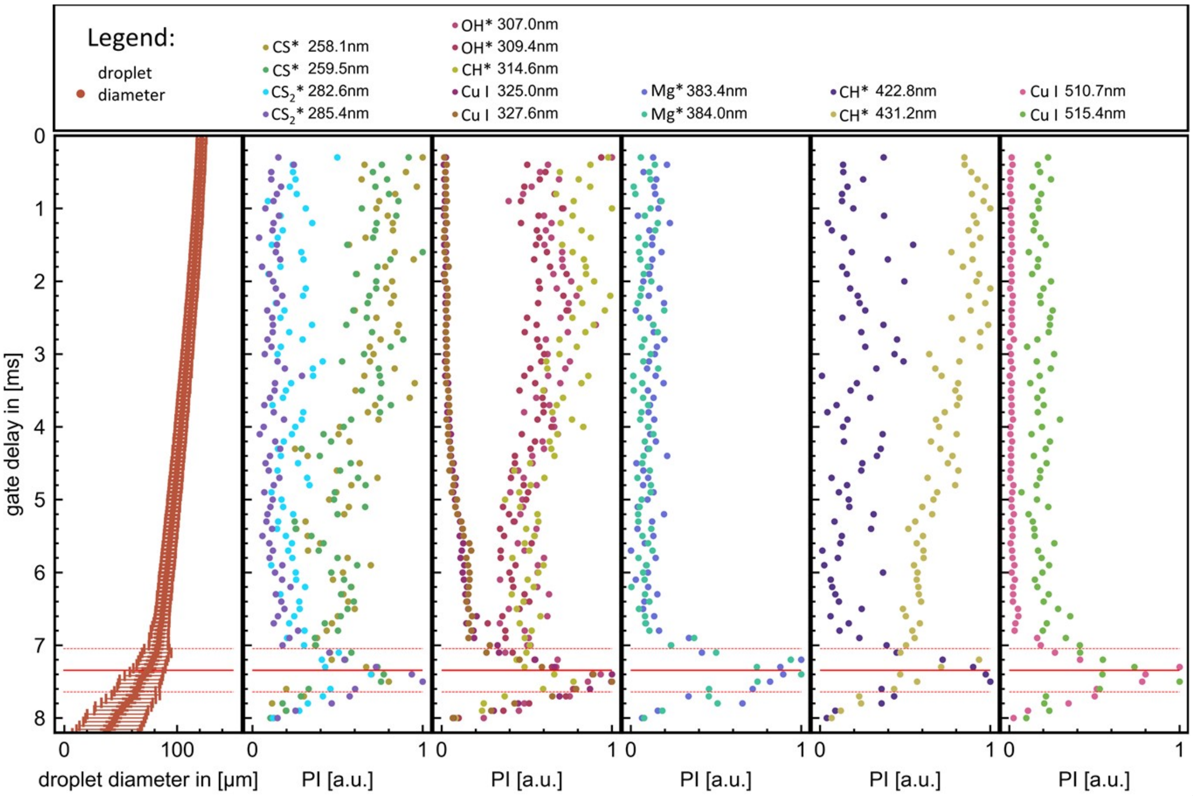


*Figure 8 plot of the highspeed imaging results and temporal resolved emission peaks from flame emission spectroscopy for P3. The ignition of the droplet is at gate delay 0 ms coinciding with the ignition spark.*

The combustion pattern of P3 shown in Figure 8 exhibits two distinct segments before the onset of the first micro explosion at 7.345 ± 297 [ms]. The first segment is characterized by an increasing burn rate, reaching a plateau at kmax 1.23[mm2/s]. The second segment the second segment starting shortly before the micro explosion onset is characterized by a brief decrease in burn rate, followed by a subsequent increase. A second micro explosion is observed shortly after the first micro explosion.

In P3 CH, OH, and CS emissions exhibit a similar pattern - a sharp decrease followed by a peak at the onset of micro-explosion. Due to increased scattering, observations in these spectra are uncertain. The Cu peaks at 325.0 and 327.6 rises steadily after ≈ 4 ms, transitioning to a plateau/slowe rise, culminating in a strong peak coinciding the micro-explosion. The Cu peak at 510.7 nm shows a strong peak coinciding with the micro explosion. Similarly, the CS2 emission shows a strong peak coinciding with the micro-explosion, a smaller emission just before the micro-explosion is observed.

P4:

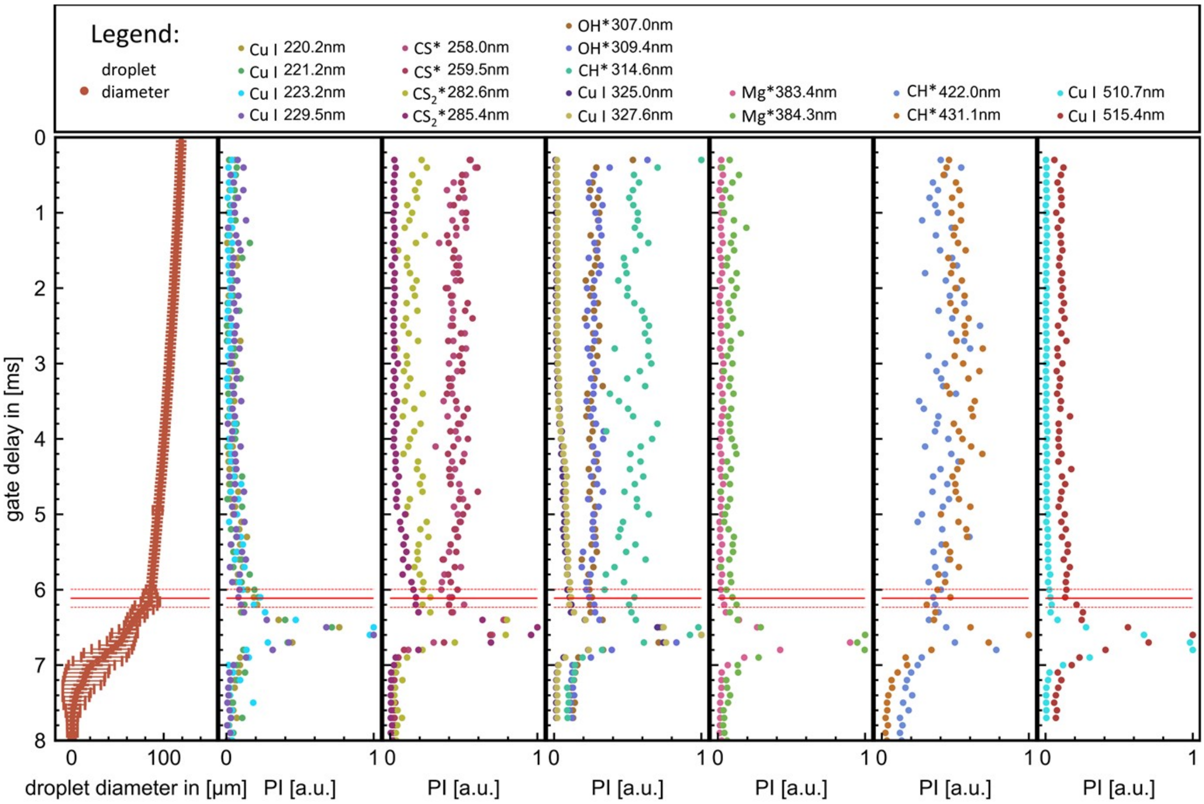


F igure 9 plot of the highspeed imaging results and temporal resolved emission peaks from flame emission spectroscopy for P4. The ignition of the droplet is at gate delay 0 ms coinciding with the ignition spark.

The combustion pattern of P4 shown in Figure 9 exhibits two distinct segments before the onset of the first micro explosion at 6.114 ± 0.120 [ms]. The first segment is characterized by an increasing burn rate, reaching a maximum of 1.38 [mm2/s]. The second segment starting shortly before the micro explosion onset is characterized by a brief decrease in burn rate, followed by a subsequent increase. A second micro explosion is observed shortly after the first micro explosion.

P4 exhibits a similar emission pattern for CH, OH and CS, with a near constant emission after ignition and a brief decline before a pronounced peak coinciding with the micro-explosion. The copper peaks at 325.0 nm and 327.6 nm consistently rise from ≈ 3.5 ms onwards, transitioning into a plateau or a slower rise before forming a strong peak coinciding with the micro-explosion. The Cu peaks at 510.7 nm shows a strong peak coinciding with the micro-explosion. Copper peaks at 225 nm are detectable shortly before the micro explosion and peak strongly with the micro explosion. CS2 emissions increases from ≈ 4.5 ms and peaks during the micro explosion.

Plot of spatial resolved flame emission spectroscopy spectra at different positions.

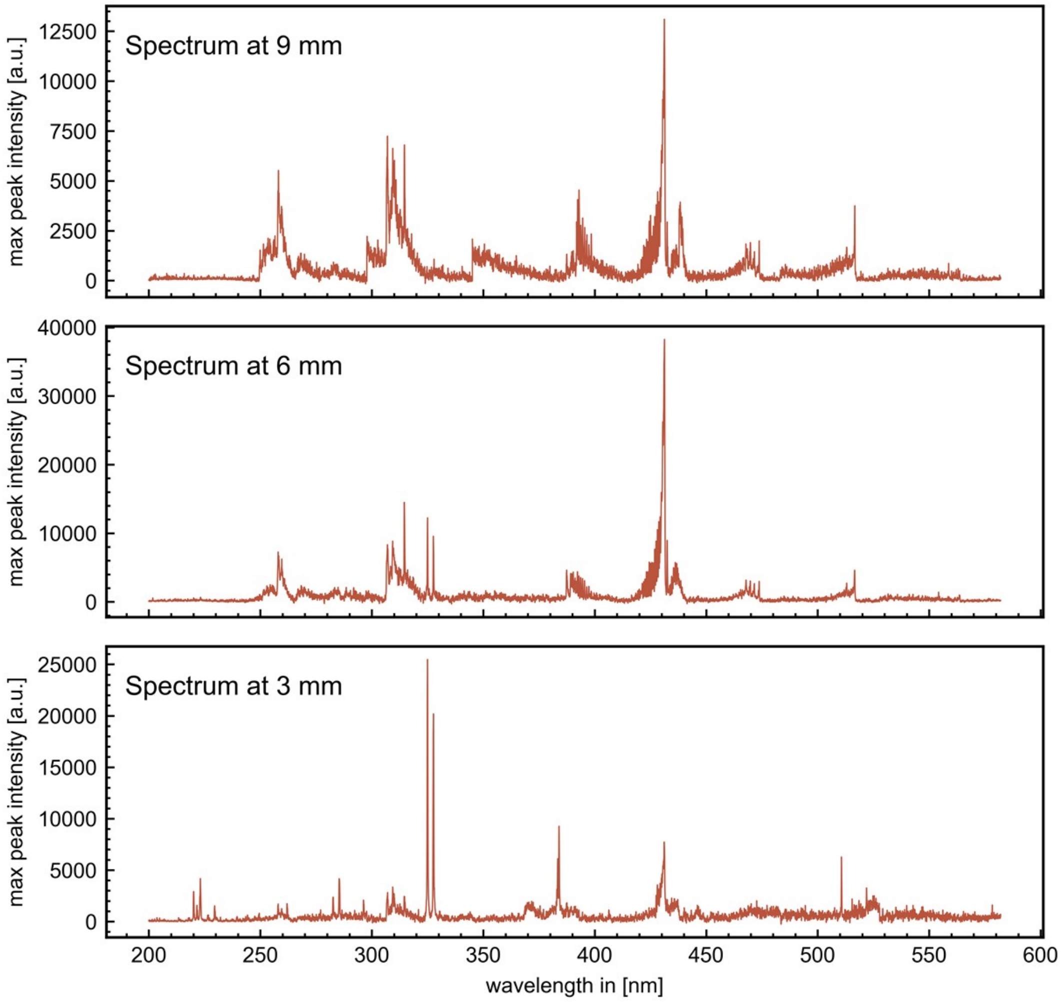


*Figure 10 Temporal resolved spectra taken from P1 at 9 mm, 6 mm, and 3 mm from top to bottom. Each spectrum was isolated for evaluation.*

Plot of temporal resolved flame emission spectroscopy spectra at different delay time steps.

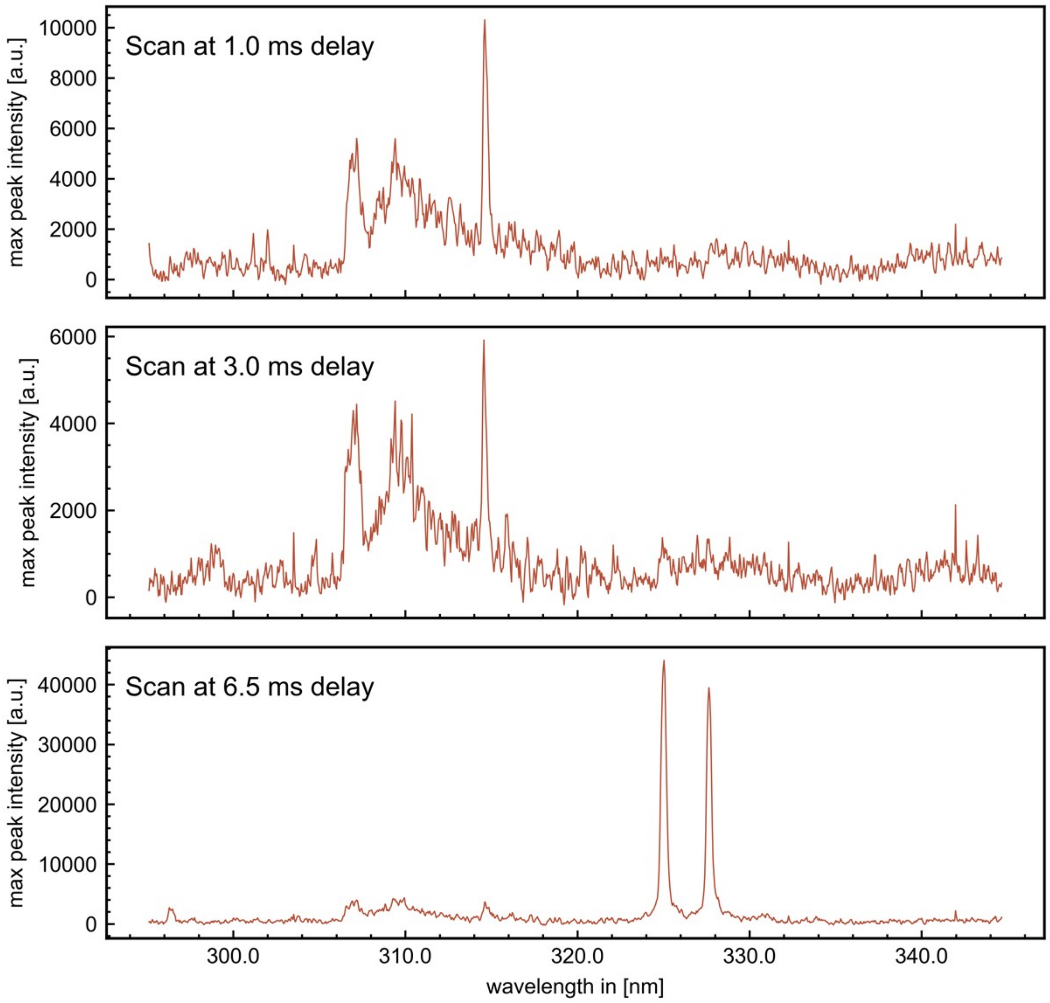


*Figure 11 Delay scan spectra from P4 taken at delay times 1.0, 3.0, and 6.5 ms, from top to bottom, to depict isolated spectra for evaluation.*

## Photograph of the experimental setup

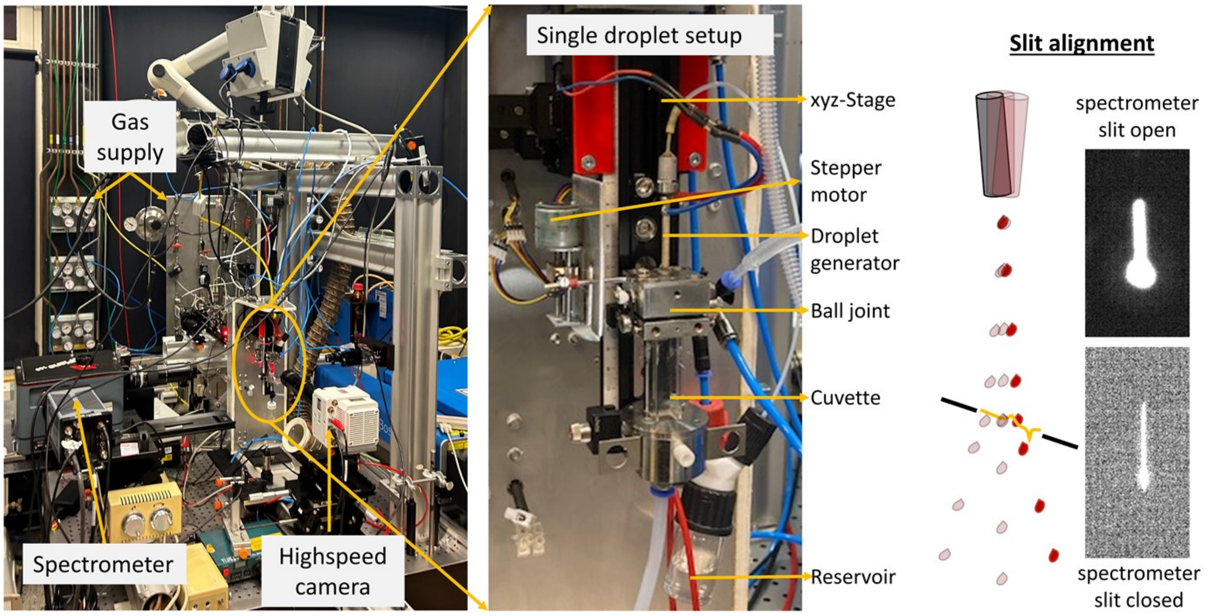


*Figure 12 Left photograph of the entire setup. Middle photograph of the single droplet reactor up close. Right depiction of the slit alignment of the droplet combustion trajectory with the slit.*
